# Optimization of Gamma Image Quality Through Experimental Evaluation Using 3D-Printed Phantoms Across Energy Window Levels

**DOI:** 10.3390/bioengineering12111211

**Published:** 2025-11-06

**Authors:** Chanrok Park, Joowan Hong, Min-Gwan Lee

**Affiliations:** Department of Radiological Science, College of Health Science, Eulji University, 553, Sanseong-daero, Sujeong-gu, Seongnam-si 13135, Republic of Korea; jwhong@eulji.ac.kr (J.H.); mglee2@g.eulji.ac.kr (M.-G.L.)

**Keywords:** electromagnetic wave, gamma camera, energy window level, 3D printing technology, quantitative analysis

## Abstract

Energy window selection is a critical parameter for optimizing planar gamma image quality in nuclear medicine. In this study, we developed dedicated nuclear medicine phantoms using 3D printing technology to evaluate the impact of varying energy window levels on image quality. Three types of phantoms—a Derenzo phantom with six different sphere diameters, a modified Hoffman phantom incorporating lead for attenuation, and a quadrant bar phantom with four bar thicknesses constructed from bronze filament—were fabricated using Fusion 360 and an Ultimaker S5 3D printer with PLA and bronze-based materials. Planar images were acquired using 37 MBq of Tc-99m for 60 s at energy windows centered at 122, 140, and 159 keV. Quantitative assessments included contrast-to-noise ratio (CNR), coefficient of variation (COV), peak signal-to-noise ratio (PSNR), and structural similarity index measure (SSIM), comparing all images with the 140 keV image as the reference. The results showed a consistent decline in image quality at 122 keV and 159 keV, with the highest CNR, lowest COV, and optimal PSNR/SSIM values obtained at 140 keV. In visual analysis using the quadrant bar phantom, thinner bars were more clearly discernible at 140 keV than at other energy levels. These findings demonstrate that the application of an appropriate energy window—particularly 140 keV for Tc-99m—substantially improves image quality in planar gamma imaging. The use of customized, material-specific 3D-printed phantoms also enables flexible, reproducible evaluation protocols for energy-dependent imaging optimization and quality assurance in clinical nuclear medicine.

## 1. Introduction

Nuclear medicine imaging entails injecting patients with radioisotopes to obtain anatomical and functional information [1,2]. Adjusting acquisition parameters in nuclear medicine imaging can improve diagnostic accuracy [3]. Planar imaging, a common technique in nuclear medicine examinations such as bone scans, renal studies, and hepatobiliary scans, often involves using two or three detectors in a gamma camera. Furthermore, the Tc-99m radioisotope is commonly preferred due to the low gamma energy (140 keV), short physical half-life (6 h), and high photon emission rate of the Tc-99m radioisotope [4]. Planar imaging in nuclear medicine captures photon distributions, such as gamma rays emitted by patients. Photon–material interactions, including photoelectric and Compton scatter effects, occur when using the Tc-99m radioisotope [5,6]. Determining the energy window for the photo peak interval of each radioisotope, typically within a ±10~20% band width of the photo peak, is crucial for correcting and optimizing gamma rays [7]. For example, the energy range for Tc-99m, with a photopeak energy of 140 keV, typically spans from 126 to 159 keV within a ±10% energy band width. Numerous studies have aimed to improve image quality by adjusting the energy window range [8,9,10]. Can et al. reported that energy window-based scatter correction techniques improved the quantitative accuracy of Tc-99m imaging [8]. However, experimental evaluations using customized 3D-printed phantoms to determine the optimal energy window remain limited. Additionally, a previous study optimized the collimator and energy window settings for Y-90 bremsstrahlung SPECT imaging, emphasizing the critical role of energy window selection in image quality [9]. Although the radionuclide differs, this finding supports the relevance of energy window optimization in Tc-99m imaging, as investigated in our study. Another related study examined the optimization of energy windows in bremsstrahlung imaging after Y-90 microsphere therapy, focusing on the trade-off between scatter reduction and system sensitivity. However, that investigation was limited to clinical and computational approaches and did not include experimental validation using physical phantoms [10].

In clinical practice, gamma cameras are subject to routine quality assurance (QA) and quality control (QC) procedures to ensure consistent diagnostic performance. The ability to objectively assess image quality based on energy window settings is essential for maintaining high standards in nuclear medicine imaging. Using a dedicated nuclear medicine phantom, QC and QA procedures are performed before and after scanning patients to mitigate the risk of degraded image quality and inaccurate diagnostic results [11]. These procedures involve acquiring images of the nuclear medicine phantom by administering a relative radioisotope, tailored to the scanning objective. Typically, evaluations involve spatial resolution, uniformity, signal-to-noise ratio (SNR), and contrast-to-noise ratio (CNR). For instance, the Derenzo phantom enables the acquisition of images featuring various sphere sizes, facilitating comprehensive assessments [12]. Furthermore, the Hoffman phantom proves valuable for imaging brain lesions or soft tissue, while the quadrant bar phantom allows for spatial resolution assessment through visual confirmation of four different bar thicknesses [13,14]. Although standard nuclear medicine phantoms offer numerous advantages in terms of image quality, geometry is difficult to modify for diverse experimental purposes. To address this problem, many researchers have developed customized nuclear medicine phantoms. Commercially available phantoms, such as the Jaszczak and Hoffman models, have been widely used for quality assurance and system calibration in nuclear medicine imaging. However, their fixed geometries, limited material options, and high manufacturing costs restrict their adaptability to specific experimental conditions. These constraints have motivated the development of customizable alternatives capable of representing diverse anatomical or physical configurations at lower cost. Berthon et al. reported on a novel phantom that effectively improved the accuracy of automatic segmentation based on positron emission tomography (PET) within a radiotherapy treatment planning system [15]. Similarly, Markievicz et al. suggested an approach to improve quantitative analysis and spatial resolution using a customized phantom [16]. Additive manufacturing, commonly known as 3D printing, has emerged as a practical solution for producing customized nuclear medicine phantoms [17,18,19]. The technology allows for the rapid and precise fabrication of phantoms with complex internal geometries, adjustable densities, and material-specific attenuation characteristics. Such flexibility is particularly advantageous when tailoring phantom structures to specific imaging purposes or experimental setup. Moreover, the ability to incorporate materials with varying radiation interaction properties enables researchers to simulate clinically relevant scenarios with improved fidelity. These advantages collectively reduce production costs and time while enhancing experimental reproducibility. In light of these strengths, 3D printing has gained increasing attention as a platform for the development of application-specific phantoms in nuclear medicine imaging. 3D printing-based phantoms offer efficiency, cost-effectiveness, and flexibility in determining geometry, making them a favorable option. Kiss et al. fabricated an anatomically realistic left ventricular myocardial phantom using 3D printing technology to evaluate nuclear medicine imaging performance under controlled conditions [20]. However, this study primarily focused on cardiac anatomy and did not evaluate the impact of acquisition parameters such as energy window settings, leaving a gap in the comprehensive assessment of image quality optimization using variable acquisition conditions. In addition, Läppchen et al. demonstrated the feasibility of fabricating radioactive phantoms using 3D printing to simulate clinical nuclear medicine imaging [21]; however, this study did not explore energy window optimization or conduct a systematic assessment of image quality. Bieniosek et al. proved and suggested that the 3D printing imaging phantom they developed was compatible with multiple modalities, including PET/computed tomography (CT) and PET/magnetic resonance imaging (MRI) [22]. In a previous study, our research team evaluated gamma image quality by manufacturing a customized 3D printing phantom using polylactic acid (PLA) (density: 1.25 g/cm^3^) and copper (density: 8.96 g/cm^3^) [20]. In recent years, the use of 3D printing technology in crafting nuclear medicine phantoms has seen a gradual rise.

Therefore, the aim of this study was to comprehensively evaluate the impact of different energy window settings on the image quality of Tc-99m gamma planar imaging by employing a series of quantitative and visual assessments. To this end, we utilized a set of customized nuclear medicine phantoms—including Derenzo, Hoffman, and quadrant bar phantoms—fabricated via 3D printing to enable controlled and reproducible experimental conditions.

## 2. Materials and Methods

### 2.1. Dedicated 3D Printing Nuclear Medicine Phantom

**Figure 1:**
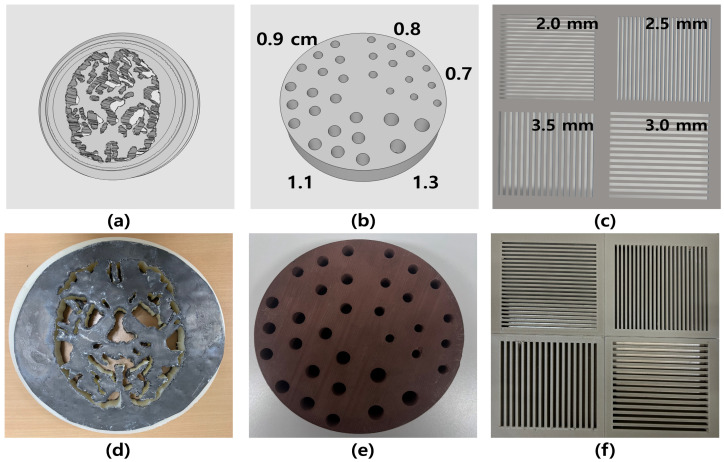
Designs of (**a**) Hoffman, (**b**) Derenzo (0.7–1.3 cm spheres), and (**c**) quadrant bar (2.0–3.5 mm thickness) phantoms created using Fusion 360 software; (**d**–**f**) corresponding 3D-printed phantoms, respectively.

Unlike previous designs that focused solely on basic geometric structures, the present study introduced newly developed 3D-printed phantoms that incorporated enhanced complexity and material diversity. The quadrant bar phantom and modified Hoffman phantom were fabricated with adjustable thicknesses and embedded with high-density materials, such as lead and bronze, to simulate clinically relevant attenuation effects. Except for the Derenzo phantom, which had been evaluated in a prior study, all phantom designs were newly constructed and optimized to support detailed assessment of energy-dependent image quality in planar gamma imaging [21]. These custom phantoms provided greater experimental flexibility and more realistic imaging conditions compared to commercially available or previously published models. Dedicated 3D phantoms for nuclear medicine were printed using an Ultimaker S5 printer, employing various filament materials. Fusion 360 software (Autodesk https://www.autodesk.com/) was used to generate the blueprint and STL files. Subsequently, the generated STL file was converted into a G-code file using Cura software (version 5.4.0) to facilitate the printing of the nuclear medicine phantoms. In this study, the designs and structures of the fabricated phantoms are presented in Figure 1. We designed and printed a Hoffman phantom with a diameter of 15 cm by converting computed tomography images into an STL file through 3D slicer software (version 5.8.1). The phantom was constructed using PLA filament material, as shown in Figure 1a. Furthermore, to attenuate the transmission of gamma rays, lead material (density: 11.34 g/cm^3^) was incorporated into the printed Hoffman phantom, as shown in Figure 1d. In a previous study, the Derenzo phantom had a diameter of 15 cm and included various sphere sizes ranging from 0.7 and 1.3 cm, as shown in Figure 1b,e [23]. Additionally, a quadrant bar phantom, constructed from bronze filament material (density: 8.73 g/cm^3^), was manufactured with dimensions of 280 × 215 mm^2^. This phantom comprised 22 bars with a thickness of 2.0 mm, 23 bars with a thickness of 2.5 mm, 15 bars with a thickness of 3.0 mm, and 17 bars with a thickness of 3.5 mm as shown Figure 1c,f.

### 2.2. Image Acquisition Parameters

**Figure 2:**
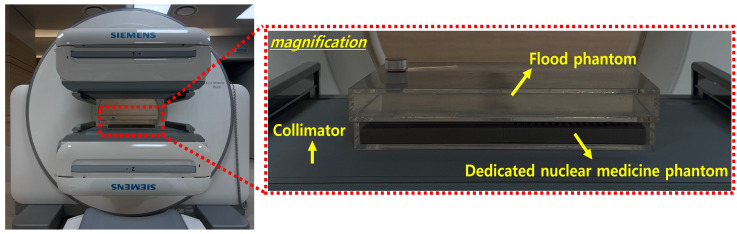
Experimental setup featuring a gamma camera equipped with a low-energy high-resolution (LEHR) collimator and NaI(Tl) scintillator crystal, along with a flood phantom containing Tc-99m, and dedicated nuclear medicine phantoms fabricated using 3D printing techniques.

Prior to image acquisition, daily quality control procedures, including uniformity and energy calibration verification using a Tc-99m flood source, were performed following the manufacturer’s standard protocol. The system dead time was confirmed through count rate linearity tests. The experiment utilized a dual-headed gamma camera (Symbia Intevo Bold SPECT; Siemens, Munich, Germany). Figure 2 shows the experimental setup, which includes the low-energy high-resolution (LEHR) collimator and a NaI(Tl) scintillation crystal with dimensions of 59.1 cm × 44.5 cm and a thickness of 0.9525 cm. The LEHR collimator features hexagonal holes with a length of 2.405 cm and a septal thickness of 0.016 cm. Additionally, a flood phantom injected with 37 MBq of Tc-99m (140 keV) was positioned on the detector. Hoffman, Derenzo, and quadrant bar phantoms were positioned, and images of each phantom were acquired for 60 s under equal-time conditions without count normalization. The energy window was adjusted to 122 keV ± 10%, 140 keV ± 10%, and 159 keV ± 10%, respectively, so that variations in photon counts reflected intrinsic differences between the photopeak and scatter fractions rather than inconsistencies in system performance. Furthermore, a flood phantom image was acquired to evaluate uniformity according to the applied energy window. Planar image of each phantom was obtained using one detector of the gamma camera, with a matrix size of 256 × 256, a zoom factor of 2.0, and a pixel size of 2.39 × 2.39 mm^2^. Each experiment was repeated five times independently to minimize random variations and to ensure statistical reliability.

### 2.3. Quantitative Analysis

**Figure 3:**
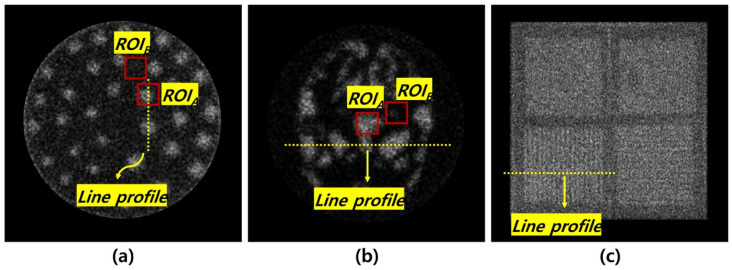
Planar images of (**a**) Derenzo, (**b**) Hoffman, and (**c**) quadrant bar phantoms; ROI_A_ and ROI_B_ used for contrast to noise ratio (CNR) calculation, ROI_A_ for coefficient of variation (COV), and dashed line profiles for intensity analysis.

To evaluate the image quality, both traditional and similarity-based metrics were employed. The CNR and coefficient of variation (COV) were calculated using regions of interest (ROIs) to assess image contrast and uniformity, respectively. As shown in Figure 3, ROI_A_ and ROI_B_ were used for CNR calculation, while ROI_A_ was used for COV assessment. Additionally, intensity profile analysis was conducted along selected lines passing through key structures in each phantom as shown Figure 3. In addition, quantitative similarity analyses were performed using peak signal-to-noise ratio (PSNR), structural similarity index measure (SSIM) with the 140 keV image used as the reference standard. These metrics provided complementary insights into the structural fidelity and intensity-based differences among the images acquired at 122 keV, 140 keV, and 159 keV. The formulas for each are as follows:

(1) $$CNR=\;\frac{\left|\left.M_{H}-M_{B}\right|\right.}{\sqrt{\sigma_{H}^{2}+\sigma_{B}^{2}}}$$

(2) $$COV=\frac{\sigma_{H}}{M_{H}}$$

where $M_{H}$ and $\sigma_{H}$ represent the mean pixel value and standard deviation of the set ROIs, while $M_{B}$ and $\sigma_{B}$ denote the background values of mean pixel and standard deviation, respectively. This study specifically compared the image quality using the Derenzo phantom for a sphere size of 1.1 cm and the Hoffman phantom. The PSNR and SSIM are calculated as

(3) $$MSE=\frac{1}{mn}\sum_{i=1}^{m}\sum_{j=1}^{n}{\left(I\left(i,j\right)-K\left(i,j\right)\right)}^{2}$$

(4) $$PSNR=10\cdot \log_{10}\left(\frac{{MAX}_{I}^{2}}{MSE}\right)$$

where m × n  denotes the image dimensions, and $I\left(i,j\right)\;$ and $K\left(i,j\right)$ represent the pixel intensities of the reference and comparison images at position $\left(i,j\right)$, respectively. In addition, ${MAX}_{I}$ is the maximum possible pixel value of the image, set to 255 in this study for 8-bit grayscale images, and MSE is the mean squared error between reference and comparison images.

(5) $$SSIM=\frac{\left(2\mu_{I}\mu_{K}+C_{1})(2\sigma_{IK}+C_{2}\right)}{\left(\mu_{I}^{2}+\mu_{K}^{2}+C_{1})(\sigma_{I}^{2}+\sigma_{K}^{2}+C_{2}\right)}$$

where $\mu_{I}$ and $\mu_{K}\;$ denote the mean intensities of the reference and evaluated images, respectively; $\sigma_{I}^{2}\;$ and $\sigma_{K}^{2}\;$ are the corresponding variances; and $\sigma_{IK}$ is the covariance between the two images. Constants $C_{1}\;$ and $C_{2}\;$ are small values introduced to stabilize the division in cases of weak denominators.

## 3. Results

**Figure 4:**
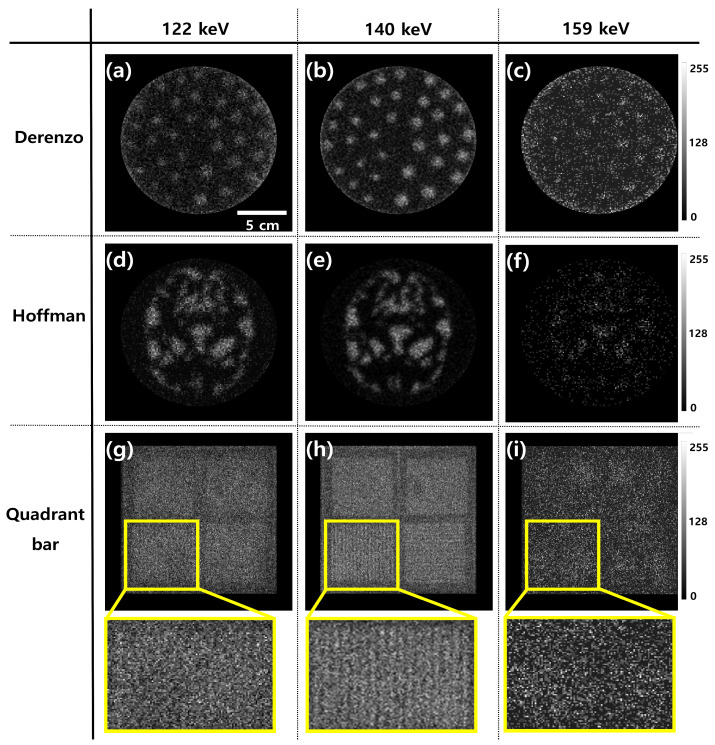
Acquired images showcasing (**a**–**c**) Derenzo phantoms, (**d**–**f**) Hoffman phantoms, and (**g**–**i**) quadrant bar phantoms, including magnified views of the 3.5 mm bar thickness, captured at energy levels 122, 140, and 159 keV, respectively, all of which include a 5 cm scale bar and are displayed with a consistent color map representing count distributions normalized to the maximum pixel intensity of each image.

**Figure 5:**
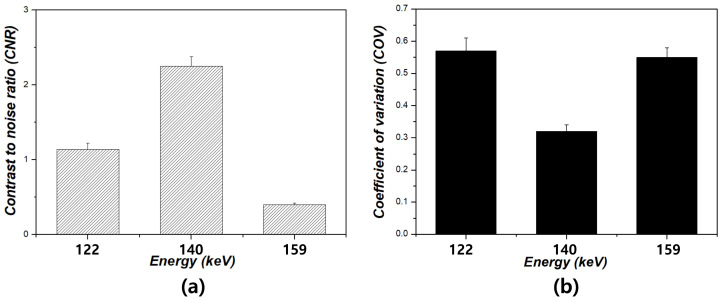
Results of (**a**) contrast-to-noise ratio (CNR) and (**b**) coefficient of variation (COV) measured from the 1.1 cm spheres of the Derenzo phantom under energy windows of 122, 140, and 159 keV.

**Figure 6:**
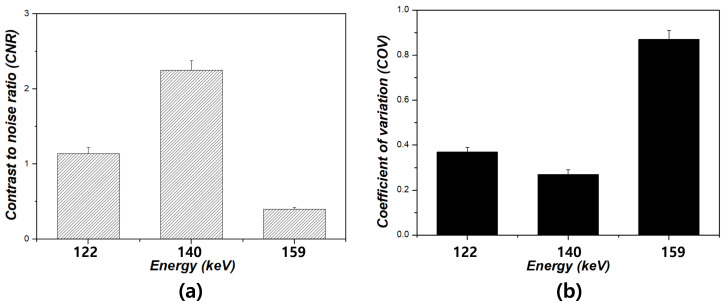
Results of (**a**) contrast-to-noise ratio (CNR) and (**b**) coefficient of variation (COV) for the Hoffman phantom under energy windows of 122, 140, and 159 keV.

Figure 4 shows the resulting images corresponding to the energy window level settings for each phantom. Figure 4a–c show the Derenzo phantom images at 122, 140, and 159 keV, respectively. Notably, the images at 159 keV showed no distinguishable features. Furthermore, the sphere size was clearly discernible at 140 keV compared to that at 122 and 159 keV. In the Hoffman phantom images, superior image quality was observed at 140 keV. When examining the quadrant-bar phantom, magnified images with a 3.5 mm bar thickness were distinguishable under the 140 keV condition, contrasting with the images obtained at 122 and 159 keV. Figure 5 shows the resulting graph depicting the energy conditions of 122, 140, and 159 keV at a sphere size of 1.1 cm for the Derenzo phantom. The CNR values were 1.14 ± 0.08, 2.25 ± 0.13, and 0.40 ± 0.02 under the energy conditions of 122, 140, and 159 keV, respectively. Based on the results, the CNR value at the condition of 140 keV was the highest compared to the other scan conditions. Specifically, the CNR value at the energy condition of 140 keV was approximately 1.97 higher than that at 122 keV and 5.59 times higher than that at 159 keV. Additionally, the COV values were 0.59 ± 0.04, 0.32 ± 0.02, and 0.55 ± 0.03 under the energy conditions of 122, 140, and 159 keV, respectively. The COV value under the energy condition of 140 keV was approximately 1.83 times lower than that under 122 keV and 1.7 times lower than that under 159 keV. The CNR and COV results for the Hoffman phantom are presented in Figure 6. According to the results, the CNR values were 2.10 ± 0.13, 2.97 ± 0.16, and 0.94 ± 0.05 under the energy conditions of 122, 140, and 159 keV, respectively. Additionally, the CNR under the energy condition of 140 keV was approximately 1.83 times higher than that under 122 keV and 1.71 times higher than that under 159 keV, respectively. Regarding the COV values, they were 0.37 ± 0.02, 0.27± 0.02, and 0.87 ± 0.04 for the energy conditions of 122, 140, and 159 keV, respectively. Furthermore, the COV result for the energy condition of 140 keV was approximately 1.34 times lower than that for 122 keV and 3.25 times lower than that for 159 keV.

**Figure 7:**
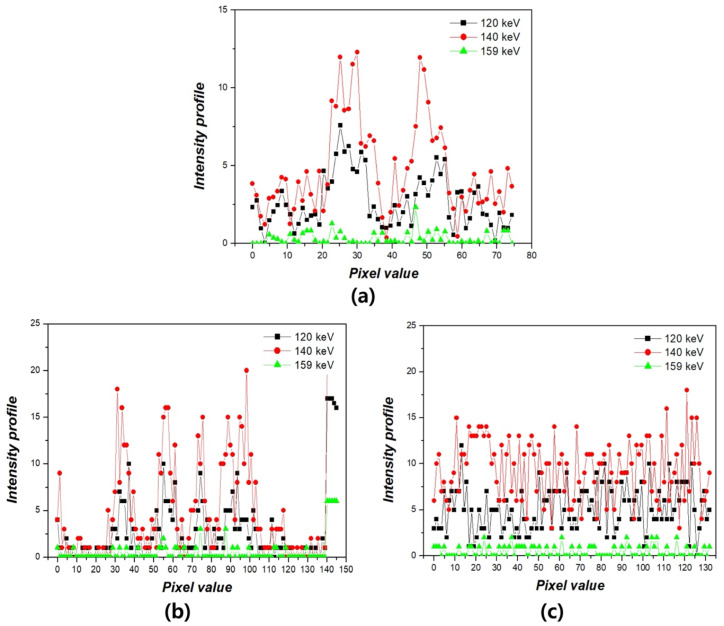
Intensity profile analysis of gamma images acquired at three energy windows (122 keV, 140 keV, and 159 keV) using (**a**) Derenzo phantom, (**b**) Hoffman phantom, and (**c**) Quadrant bar phantom.

**Figure 8:**
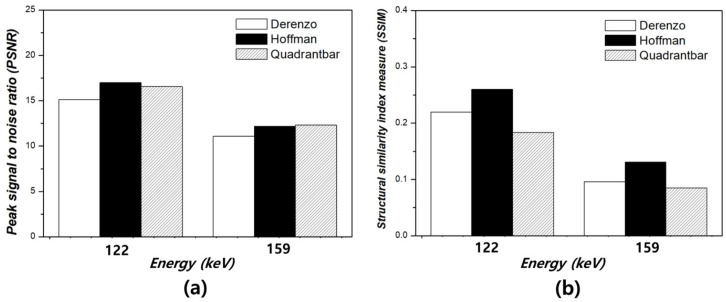
Quantitative similarity analysis using (**a**) peak signal-to-noise ratio (PSNR) and (**b**) structural similarity index measure (SSIM) between the reference gamma images acquired at 140 keV and the compared images acquired at 122 keV and 159 keV for three different phantoms: Derenzo, Hoffman, and Quadrant bar.

To further evaluate the image quality according to energy window levels, intensity profiles were extracted from three phantoms—Derenzo, Hoffman, and Quadrant bar—as shown in Figure 7. The profiles were measured along a consistent axis in each phantom to assess the relative variation in pixel intensities across structural boundaries. In the Derenzo phantom, the profile corresponding to the 140 keV condition showed the most distinct and repetitive peaks, reflecting the presence of clearly resolved spherical inserts of varying diameters. These rapid fluctuations in intensity indicate a well-preserved edge definition and spatial resolution. In contrast, the 159 keV image exhibited a markedly flattened intensity curve, indicating a substantial loss of structural information and edge degradation. The 122 keV image showed intermediate behavior, with detectable but less pronounced intensity variations. For the Hoffman phantom, the intensity profile at 140 keV revealed sharp transitions between adjacent anatomical regions, suggesting improved contrast and boundary visibility between grey and white matter analogues. On the other hand, profiles from 122 keV and 159 keV displayed smoother transitions and attenuated peaks, reflecting diminished anatomical delineation and lower contrast fidelity. The Quadrant bar phantom, designed for evaluating spatial resolution, further demonstrated the superiority of the 140 keV energy window. The 140 keV profile distinctly resolved each bar pattern, even at smaller thicknesses, through a periodic intensity fluctuation. In contrast, the 159 keV profile was nearly flat, implying insufficient contrast to differentiate individual bars, while the 122 keV profile again showed modest but insufficient edge definition. These findings collectively support the conclusion that 140 keV offers optimal performance for edge preservation and structural fidelity in Tc-99m planar gamma imaging. The sharpness of the intensity transitions at 140 keV demonstrates a superior ability to resolve anatomical and geometric details, which is crucial for accurate clinical interpretation. This underscores the critical importance of selecting an appropriate energy window when acquiring planar gamma images, as deviations from the photopeak energy result in noticeable degradation of image sharpness and diagnostic quality. Figure 8a,b present the PSNR and SSIM results for the three phantoms (Derenzo, Hoffman, and Quadrant bar) under energy windows of 122 keV, 140 keV, and 159 keV, with the 140 keV image used as the reference. Given that 140 keV corresponds to the clinical photopeak of Tc-99m and is widely adopted in standard protocols, it was used as the reference baseline. For the Derenzo phantom, the PSNR was 15.13 ± 0.20 dB at 122 keV and 11.08 ± 0.15 dB at 159 keV, while SSIM was 0.220 ± 0.003 and 0.096 ± 0.001, respectively. For the Hoffman phantom, PSNR values were 16.99 ± 0.15 dB (122 keV) and 12.19 ± 0.12 dB (159 keV), and SSIM values were 0.260 ± 0.003 and 0.131 ± 0.002. For the Quadrant bar phantom, PSNR was 16.55 ± 0.10 dB at 122 keV and 12.30 ± 0.10 dB at 159 keV, with corresponding SSIM values of 0.183 ± 0.002 and 0.085 ± 0.001. Overall, both metrics consistently indicated a noticeable degradation in image quality at energy windows of 122 keV and 159 keV compared to the 140 keV reference. PSNR and SSIM values at 122 keV and 159 keV were notably reduced, suggesting that both lower and higher energy windows lead to perceptible loss in image quality.

## 4. Discussion

The Tc-99m radioisotope, derived from the generator, is widely used in nuclear medicine imaging. Optimizing the acquisition of gamma rays emitted by patients is crucial for enhancing image quality in nuclear medicine imaging. To enable quantitative analysis independent of conventional geometries, customized 3D printing phantoms designed for acquiring gamma planar images have been developed. These phantoms are fabricated using appropriate filament materials to ensure effective transmission protection against gamma rays. Previous work by Ferreira et al. focused on the development of an anthropomorphic liver phantom specifically designed for quality control and training applications in nuclear medicine [24]. The phantom incorporated cold and hot regions with varying sizes and positions, enabling routine assessment of gamma camera performance and technician training in image interpretation. Constructed from acrylic and plaster materials, the phantom allowed for reproducible evaluation of imaging parameters under standardized conditions. In contrast to this conventional fabrication approach, the present study utilized 3D printing technology to design and produce dedicated planar imaging phantoms—including Derenzo, Hoffman, and quadrant bar models—with fine structural details and customizable internal geometries. This method allowed for the precise modulation of physical dimensions and material density, which are critical for simulating clinically relevant uptake patterns in Tc-99m imaging. While conventional metrics such as CNR and COV are informative for overall contrast and variability, they do not fully capture subtle structural distortions or localized image degradation. The use of PSNR and SSIM in this study provided critical insight into the structural fidelity and noise characteristics of gamma images acquired under varying energy windows. These findings reinforce the critical importance of selecting an appropriate energy window in nuclear medicine imaging. Even slight deviations from the optimal photopeak-centered window (e.g., 140 keV for Tc-99m) resulted in significant degradation of image quality, as evidenced by PSNR and SSIM metrics across all phantom types. This suggests that precise energy window calibration is not merely a technical setting, but a pivotal factor directly influencing diagnostic reliability, especially in clinical and QA protocols.

In addition to demonstrating the impact of energy window selection on gamma image quality, our research highlights the practical benefits of using 3D printing techniques for phantom fabrication in nuclear medicine. The customized phantoms developed in this study offered structural flexibility that is difficult to achieve with commercially available models, allowing for precise control over geometry, size, and material properties. Moreover, the use of cost-effective materials and rapid prototyping significantly reduced production time and expenses, allowing the fabrication of phantoms with diverse internal structures tailored to specific imaging objectives. Thus, these advantages suggest that 3D printing can serve as a robust alternative to conventional phantom production, especially when experimental customization and resource efficiency are essential. In addition, conventional nuclear medicine phantoms such as Jaszczak have long served as benchmarks for system calibration, uniformity evaluation, and spatial resolution assessment in nuclear medicine imaging. However, these commercially manufactured phantoms often possess fixed internal geometries and lack adaptability for task-specific imaging evaluation. In contrast, the 3D-printed phantoms developed in this study were designed with customizable configurations, enabling precise control over object density, rod spacing, and structural complexity. This flexibility allows researchers to simulate a wide range of clinical imaging study, including non-standard energy windows and novel detector geometries. While the proposed phantom system may not aim to replace existing standardized models, proposed phantom offers unique advantages in terms of cost-effectiveness, rapid prototyping, and geometric versatility. These characteristics make the developed phantom system particularly suitable for iterative experimental studies, training purposes, and QA protocol development tailored to emerging radionuclides or imaging technologies.

One limitation of the present study is the exclusive use of Tc-99m, a radionuclide with well-established physical characteristics and imaging performance. Although the selection of Tc-99m ensured consistency across imaging conditions and clinical relevance, the applicability of the 3D-printed phantom system under varying photon energies remains unverified. To address this, subsequent investigations should assess phantom performance using other clinically utilized radionuclides, including I-123, In-111, and Lu-177, which emit gamma photons at different energy levels. Evaluating image quality across a wider range of radionuclides would contribute to a more comprehensive validation of the phantom’s structural flexibility and material responsiveness. Incorporating diverse isotopes could also support the development of energy window settings optimized for the unique photon interactions of each radionuclide, thereby improving the applicability of the phantom system in broader nuclear medicine imaging environments. Although the current study employed standardized 3D-printed phantoms to evaluate energy window-dependent image quality, future studies may benefit from the integration of anatomically realistic, organ-specific phantom models, such as the cardiac phantom proposed by Green et al. [25]. This approach would enable the assessment of energy window optimization under more clinically relevant conditions, potentially improving the translational applicability of the findings. In future studies, we plan to perform a systematic comparison of image quality using symmetric and asymmetric energy windows centered on the 140 keV photopeak (e.g., ±7%, ±10%, and ±13%) to identify the optimal configuration for Tc-99m planar gamma imaging. Future studies will expand this work by validating the optimized energy window settings in clinical imaging data obtained from patients. To enhance the clinical interpretability of the findings, subsequent research will aim to integrate patient-based imaging data to investigate whether improvements observed in phantom images translate into better lesion detectability and diagnostic confidence in real-world environment.

## 5. Conclusions

In this study, dedicated nuclear medicine phantoms—including a modified Hoffman phantom and a quadrant bar phantom—were fabricated using 3D printing techniques. These phantoms were utilized to systematically evaluate the impact of varying energy window settings on planar gamma image quality following Tc-99m administration. Quantitative image assessments based on PSNR, SSIM, CNR, and COV were conducted to characterize structural similarity, contrast, noise properties, and signal stability under different energy conditions. The results consistently demonstrated significant degradation in image quality outside the standard 140 keV ±10% window. These findings underscore the critical importance of energy window selection as a key acquisition parameter for achieving optimal image quality and ensuring consistency in clinical and quality assurance protocols for nuclear medicine imaging.

## Data Availability

The original contributions presented in the study are included in the article, further inquiries can be directed to the corresponding author.
